# Sex Differences and the Influence of Sex Hormones on Cognition through Adulthood and the Aging Process

**DOI:** 10.3390/brainsci8090163

**Published:** 2018-08-28

**Authors:** Caroline Gurvich, Kate Hoy, Natalie Thomas, Jayashri Kulkarni

**Affiliations:** Monash Alfred Psychiatry Research Centre, Central Clinical School, Monash University and The Alfred Hospital, Melbourne, VIC 3004, Australia; kate.hoy@monash.edu (K.H.); natalie.thomas@monash.edu (N.T.); jayashri.kulkarni@monash.edu (J.K.)

**Keywords:** sex differences, sex hormones, aging, cognition

## Abstract

Hormones of the hypothalamic-pituitary-gonadal (HPG) axis that regulate reproductive function have multiple effects on the development, maintenance and function of the brain. Sex differences in cognitive functioning have been reported in both health and disease, which may be partly attributed to sex hormones. The aim of the current paper was to provide a theoretical review of how sex hormones influence cognitive functioning across the lifespan as well as provide an overview of the literature on sex differences and the role of sex hormones in cognitive decline, specifically in relation to Alzheimer’s disease (AD). A summary of current hormone and sex-based interventions for enhancing cognitive functioning and/or reducing the risk of Alzheimer’s disease is also provided.

## 1. Introduction

Hormones of the hypothalamic-pituitary-gonadal (HPG) axis that regulate reproductive function are also potent neurosteroids and have multiple effects on the development, maintenance and function of the brain [1]. Sex differences in cognitive functioning and sex differences in the vulnerability and manifestation of several psychiatric and neurological diseases that involve cognitive disruption (such as Alzheimer’s disease (AD) and schizophrenia) highlight the potential influence of sex hormones on cognitive functioning. Sex differences in the brain and behaviour depend on a range of social and biological influences, the latter including genetic and epigenetic factors, sex chromosomes, mitochondria from the mother, and sex hormones. The focus of the current review is to provide an overview of the literature on sex differences and the role of sex hormones in relation to cognitive functioning throughout the lifespan and the aging process.

## 2. Sex Hormones and Cognition

Sex steroid hormones, including estrogens, progesterone and androgens are regulated by the HPG axis. While the neuroendocrine control mechanisms are complex, a simplified account of the HPG axis suggests that the hypothalamus releases gonadotropin-releasing hormone (GnRH) which stimulates the anterior pituitary to produce and secrete gonadotropins, including luteinizing hormone (LH) and follicle-stimulating hormone (FSH), into the bloodstream. LH travels through the blood stream to stimulate the release of the sex steroids, androgens and estrogens, from the gonads. These sex steroids complete the endocrine feedback loop by inhibiting the release of GnRH. It is well known that the roles of gonadal hormones extend beyond the regulation and development of reproductive functions. Evidence accumulated over the last four decades, from animal studies, cell culture studies and studies in humans support the influence of sex hormones on brain processes involved in cognitive functioning.

There is a relatively large knowledge base, primarily derived from rodent studies, demonstrating that estrogens can facilitate higher order cognitive functioning via a range of likely interrelated functions that involve multiple signalling pathways [2]. Estrogen-regulated synapse formation and turnover is mediated via both genomic (mediated by activation of estrogen receptor ER-α and ER-β) and rapid, non-genomic mechanisms (mediated via membrane-bound ER-α and ER-β and G protein-coupled estrogen receptor 1) in cognitively relevant brain regions, including the hippocampus and prefrontal brain regions [3,4,5]. Estrogen promotes neurotrophin synthesis [6]; modulates cholinergic [7] and dopaminergic neurotransmitter [8] systems and protects the brain against stress and inflammation [9]. Animal studies have additionally provided evidence that exogenous administration of estrogen (specifically 17β-estradiol, as opposed to estrone) has the capacity to enhance cognition, particularly in the areas of learning and memory [10,11,12]. Cognitive outcomes in human studies involving estrogen-containing hormone therapies have mostly been conducted in postmenopausal women and the results have been mixed. Unopposed estradiol administration has generally been associated with positive effects on verbal working memory and attention and this benefit is thought to be mediated through prefrontal brain networks [13,14,15].

Like estradiol, progesterone exerts trophic effects on brain development throughout adolescence and adulthood [16]. Estrogens and progesterones are thought to act together to enhance neuronal function through mechanisms such as synapse formation and reduction, enhancing synaptic transmission and exerting neuroprotective effects [5,16,17,18]. Progesterone receptors have also been identified in cognitively relevant brain regions, including the frontal cortex, hypothalamus, thalamus, hippocampus, amygdala, and cerebellum [19]. Animal studies assessing the effects of progesterone administration to ovariectomized rats are not as established compared to studies in estradiol; however, results generally suggest that progestins and progesterones can have beneficial effects on spatial cognitive performance that may be dependent on the timing of administration and the type of progestin/progesterone [16]. Human studies (in postmenopausal women and contraceptive pill studies) indicate that the type of progestin is relevant to the cognitive effects [18].

Gonadotropin levels, including LH and FSH have also been connected with cognitive functioning and implicated in cognitive decline [20,21]. Endogenous LH may regulate learning and memory via LH receptors, which have been identified in cognitively relevant brain regions, such as the hippocampus [22], or indirectly via estrogen’s capacity to regulate LH activity (as proposed by [20]). Less research has been conducted exploring the link between FSH and cognition. Preliminary studies suggest a positive relationship exists between FSH and cognition, that is, higher levels of FSH have been linked to better cognition [23]. Collectively, these findings suggest that in addition to circulating gonadal hormone levels, gonadotropins may also play a role in mediating cognitive performance.

## 3. Sex Hormones and Cognition across the Lifespan: Prenatal Androgen Exposure, Puberty and Adulthood

Sex differences in cognitive functioning have been widely reported across a number of species, including healthy adults. On average, males outperform females on spatial abilities and females outperform males in verbal abilities. It is important to point out that these sex differences are based on averages and do not apply to all individuals. In addition to these differences there are many cognitive domains without sex differences (for a review on ‘gender similarities and differences’ see [24]). Societal ‘gender’ differences and biological sex differences including chromosomes and sex hormones are all likely to contribute to the observed sex differences in cognitive tasks [25,26]. While, the effect sizes are often small and limited to specific tasks [25,27], the research stemming from this area provides us with an opportunity to learn more about the potential influence of sex hormones on cognitive functioning.

The effect of sex hormones on cognition is thought to begin in utero when brain development diverges in males and females in response to androgen production. Human males have a surge in fetal testosterone between weeks 8 and 24 of gestation (as well as a less pronounced rise in testosterone approximately 3–4 months postnatally) [28]. During this prenatal period, male fetuses produce more than 2.5 times the levels of testosterone than female fetuses [29] and this is considered to be a ‘critical window’ where hormones can influence brain development, forming the foundations for cognitive functioning [28]. The most compelling evidence linking prenatal androgen exposure to cognition is in the domain of spatial abilities.

Prenatal androgens are hypothesised to influence lateralisation of brain and behaviour, with males developing a right hemisphere dominance that may aid performance on spatial tasks (given that visuospatial cognition is associated with a stronger involvement of the right hemisphere [30]). Several hypotheses have been proposed to explain how prenatal androgens contribute to right hemisphere dominance, collectively proposing that elevated prenatal exposure to testosterone contributes to a slower development of the left hemisphere with compensatory or enhanced development of the right hemisphere [26]. A meta-analysis investigating the strength of key hypotheses associating prenatal androgen exposure to brain lateralisation concluded that current evidence remains insufficient and further research is required [31]. Further support for prenatal androgens enhancing spatial abilities comes from clinical and non-clinical populations. Females with congenital adrenal hyperplasia (CAH), a condition characterised by prenatal overproduction of adrenal androgens, have a spatial ability advantage (although males with CAH have a spatial ability disadvantage, suggesting there are optimal levels of prenatal androgens) [32]. Females with male co-twins also exhibit superior spatial abilities, which may reflect in utero exposure to androgens [33]. Prenatal androgens are also thought to facilitate interest and engagement in male-type activities and behaviours and may, therefore, also effect mental rotation abilities indirectly, by influencing early childhood interests and task engagement in ‘gendered’ activities that in themselves promote spatial abilities [34,35]. Hence, the male advantage on mental rotation may be a combined effect of biological predisposition and social experience.

Following the influence of prenatal sex hormones on behavioural development, the second wave of sex hormone-dependent neural organisation is during adolescence [36]. Following the first year of life, the HPG-axis remains dormant until a resurgence of GnRH is secreted from the hypothalamus to facilitate pubertal onset (gonadarche). Schulz and colleagues [36,37] have produced a two-stage model theorising that gonadal steroid hormones can influence the developing brain during both the perinatal period and adolescence. They propose that the perinatal period and puberty (marking the onset of adolescence) are times during which gonadal hormones are elevated and play a role in brain organisation via mechanisms such as cell proliferation and survival and synapse formation and elimination. Empirical studies specifically examining how puberty and sex hormones relate to cognition and brain development are limited. In their longitudinal study of 126 adolescents (63 females), Herting and colleagues [38] have demonstrated that sex hormones, independent of age, uniquely relate to cortical and subcortical brain volume changes. The largest changes in cortical and subcortical volume were seen during early puberty, with less change during late puberty. Changes were also sex specific and region specific (for example, testosterone was related to amygdala volumes with a decreased right amygdala volume observed in boys but increased in girls). A recent review of puberty-related structural and functional neuroimaging research highlighted sex differences and the potential role of gonadal hormones in the relationship between pubertal stage and amygdala volume (and to a lesser extent hippocampal volume) [39]. Pubertal maturation was also associated with increased white matter density/volume over time particularly in frontal and temporal lobes, as well as cortico-cortical and cortico-subcortical association tracts that connect these regions [39].

Clear and consistent evidence linking pubertal hormones or pubertal timing to cognitive functioning is lacking (as reviewed by Berenbaum and Beltz [40]). Beltz and Berenbaum [41] related pubertal timing (assessed retrospectively) to cognitive abilities in 320 young adults. They demonstrated that pubertal timing was inversely related to 3-D mental rotation abilities in males; suggesting adolescence may reflect a period of brain organisation for cognition with an earlier period of testosterone exposure associated with a spatial advantage for males. Pubertal timing was not associated with cognitive performance in females. These findings again support the link between androgens, mental rotation, and sensitive periods of brain development.

For reproductive-age females, menstrual cycle studies and contraceptive pill studies provide an opportunity to further study how endogenous and exogenous levels of sex hormones may effect cognition. For naturally cycling women, the average menstrual cycle length is somewhere between 25 and 31 days [42]. Typically, the first seven days of the menstrual cycle (early follicular phase) are characterised by low serum levels of estradiol and progesterone. Estradiol levels then rise rapidly during the second week of the cycle (late follicular phase) to a pre-ovulatory estradiol surge which is followed by an LH surge representing a precursor to ovulation (marking mid-cycle). The luteal phase (often separated into early and late phases) is the time from the LH surge to menses. Progesterone levels rise during the luteal phase and peak around day 21 (in a standard 28-day cycle) and gradually fall. The dominant theory in research linking menstrual cycle phases to cognition suggests that the early follicular phase (low estrogen, low progesterone) is associated with better performance on ‘male favouring’ cognitive abilities—such as spatial abilities and phases of the cycle with increased estrogen and/or progesterone (e.g., late follicular or mid-luteal) associated with better ‘female favouring’ cognitive abilities, such as verbal fluency and verbal memory [43].

In their comprehensive review of the topic, Sundstrom Poromaa [43] identified 13 studies that assessed verbal abilities in relation to cycle phase, of which no consistent pattern could be identified in the results. Twelve studies that assessed menstrual cycle phase and mental rotation were identified and although most studies (8 out of 12) showed no effect of phase, 4 studies showed a relationship between early follicular or low estrogen phases and improved mental rotation performance. In separate studies, naturally cycling testosterone levels in males and females have been associated with mental rotation performance; however, the nature of the relationship is inconsistent across studies and appears to differ between males (who may have an inverted U-shaped association between testosterone and mental rotation) and females (who had a U-shaped association) [44]. Females with polycystic ovary syndrome (PCOS), a condition characterised by elevated testosterone levels, also perform better on mental rotation tasks than female peers [45]. Hence, current evidence does not support a significant effect of menstrual cycle phases on cognitive performance although circulating testosterone levels may relate to mental rotation abilities.

Contraceptive pill studies provide an opportunity to explore the influence of exogenous hormones on cognitive functioning in reproductive-age females. Oral contraceptives contain synthetic analogues of estrogen and progesterone. The most commonly used estrogen analogue is ethinylestradiol. The progestin component in oral contraceptives is more diverse, with varying degrees of androgen receptor activity. In our systematic review of 22 studies assessing the contraceptive pill and cognition [46], it was concluded that the oral contraceptive pill may enhance verbal memory performance (an improvement in verbal memory was associated with the contraceptive pill in two studies [47,48]). It was also concluded that the effect of the contraceptive pill on visualspatial abilities appears to depend on the androgenicity of the progestin component. Specifically, improvements in mental rotation have been associated with androgenic pill types and impairments associated with antiandrogenic pill types [49,50]. Studies have also explored how exogenous testosterone effects cognition and results differ according to dose and age of the women studied [51]. In females of a reproductive age, studies have found that single dose testosterone (at concentrations that replicate male testosterone concentrations) improves visual spatial abilities [52,53].

Collectively, prenatal and puberty-related increases in sex hormone levels appear to contribute to sex differences in cognitive functioning, with the most convincing evidence linking androgens to mental rotation abilities. Menstrual cycle study results do not provide convincing evidence to support significant cognitive changes across menstrual cycle phase. More research is needed to understand how different contraceptive pills influence cognitive functioning; however, existing evidence suggests a potential enhancing effect of the contraceptive pill on verbal abilities (potentially related to the estrogen component of the pill) as well as an effect on mental rotation that is dependent on the androgenicity of the progestin component.

## 4. Sex Hormones and Cognition across the Lifespan: Menopause

The natural transition to menopause is associated with fluctuating and eventually decreasing levels of ovarian estrogens and progesterone as well as an increase in serum FSH. Menopause can also be induced surgically in cases of medical necessity. The impact of both menopause (naturally and surgically) and aging on cognition is complex, with substantial interindividual variability [54]. In addition to the effects of the cessation of ovarian function, this variability is also likely to stem from the presence of affective disturbances, life experiences and psychosocial stress as well as genetic liability [54].

Natural menopause is often associated with subjective cognitive complaints, particularly in the area of memory [55,56]. While studies have documented that that the natural menopause transition is associated with decreases in verbal memory and verbal fluency [57], the subjective memory complaints have also been associated with objective cognitive changes in attention and working memory [55,56]. The cognitive changes that occur during menopause appear to be independent of that expected with normal aging, particularly in relation to the decline in verbal abilities [54]. Studies have also suggested that the cognitive changes are not explained by sleep disturbances or mood, but suggested to be related to changes in the HPG axis hormones [58,59].

Surgical menopause that includes the removal of ovaries, oophorectomy, has been associated with an increased risk of cognitive impairment and dementia later in life [60]. The hormonal changes associated with bilateral oophorectomy depends on the timing of oophectomy. Bilateral oopheractomy before reaching natural menopause causes an abrupt decline of estrogen as well as progesterone and testosterone. Several large-scale studies, including the Mayo Clinic Cohort Study of Oophorectomy and Aging [60]; a Danish nationwide historical cohort study [61]; and The Religious Orders Study and the Memory and Aging Study [62], consistently report an increasing risk of cognitive impairment and dementia with younger age at the time of oophorectomy. However, for women who commenced hormone therapy following premenopausal oophorectomy and continued hormone therapy until the natural age of menopause, there was not an increased risk of AD [60,63].

## 5. Sex Hormones and Cognition across the Lifespan: Aging

Age-related declines in cognition are reported across most cognitive domains [64]. Sex differences in cognitive trajectories in healthy aging have been examined across several studies with differing results [65,66,67]. In their recent longitudinal study of healthy older adults (part of the Baltimore Longitudinal Study of Aging), McCarrey and colleagues [68] confirmed existing literature suggesting baseline sex differences in cognitive functioning, as well as demonstrating sex differences in cognitive trajectories with aging. At baseline (average age 64.1–69.7 years) females outperformed males on tests of verbal cognition, including verbal memory and verbal fluency; however, there were no sex differences in verbal cognition longitudinal rates of change for the healthy aging sample. Males demonstrated superior visual spatial abilities as compared to females at baseline; however, males demonstrated steeper rates of cognitive decline on measures of visual spatial ability and psychomotor speed.

Endocrine-related changes that accompany aging may also contribute to age-related declines in cognitive functioning, particularly the observed sex differences in brain aging and neurodegenerative processes [69]. In females, estrogen as well as progesterone production decline substantially with reproductive senescence. The main forms of estrogen are estriol (which is elevated during pregnancy and not well studied in relation to cognition), 17β- estradiol (the most potent form of estrogen), 17α estradiol, and estrone [70]. Following menopause, the ratio of estadiol to estrone changes, with more estrone relative to estradiol [71]. Contrary to estrogen, testosterone in females, and its precursors commence a steady gradual decline commencing during the third or fourth decade and continue to decline with increasing age [51,72]. Testosterone can be converted via aromatase to estradiol, which exerts its actions via estrogen receptors; or via 5α-reductase to dihydrotestosterone, which exerts its effects via androgen receptors. In contrast to the steep gonadal hormone decline in females, males are thought to experience a small and progressive decline in several sex hormones, in particular testosterone and dehydroepiandrosterone, as well as an associated increase in LH, FSH, and sex hormone-binding globulin [73].

## 6. Sex Hormones and Sex Differences in Alzheimer’s Disease (AD)

For both males and females, an age-related loss of sex steroid hormones has been associated with an increased risk of cognitive decline [74]. Around 60–80% of all dementias are caused by AD and around two-thirds of those diagnosed with AD are females [75,76]. While the increased lifespans in women complicates the interpretation of sex-differences in AD prevalence estimates, it also highlights that women are spending longer in postmenopausal years. With increasing longevity over time (without a change in the average age at which menopause is experienced) women are now spending approximately one third of their life with substantially reduced levels of estradiol and progesterone (as compared to pre-menopause) [27]. The loss of ovarian hormones at menopause and the associated loss of their neuroprotective actions has been implicated in the increased female susceptibility to AD [27]. There are also some preliminary findings to suggest that an older age at menopause and/or a prolonged reproductive period (indicative of a greater lifetime exposure to female sex hormones) may be associated with higher cognitive performance and delayed cognitive decline [77].

More recently, gonadotropin levels, particularly LH, have been implicated in playing a role in cognitive decline [20,21,78]. Following menopause, there is a loss of negative feedback by estrogen on gonadotropin production which results in an increased production of LH. LH receptors are expressed in cognitively relevant brain regions that are susceptible to AD, such as the hippocampus [22] and are found at higher levels in brain regions susceptible to AD neuropathology [79,80]. Peripheral LH is inversely related to cognitive performance. Higher levels of plasma LH have been significantly associated with poorer memory recall (in men without dementia) [81], poorer cognition (as measured by the CAMCOG: Cambridge Cognitive Examination) in older women without dementia [23]; and plasma amyloid-beta in plasma [82]. Hence, evidence is growing to implicate LH in the development and progression of AD [20,23].

Women are disproportionately affected by AD—they are more likely to develop AD, have a faster rate of cognitive and functional decline following a diagnosis of AD [83] and appear to suffer significantly greater cognitive impairments after accounting for sex differences in age, education and dementia severity [84,85]. Sex dimorphism in AD pathologies have been reported in several mouse models. For example, utilising gene expression profiling on RNA isolated from hippocampal tissue in male and female mouse brains, substantial sex disparities were observed in the trajectory of aging, with female brains experiencing age-related changes earlier than male brains [86]. Transgenic mouse models have also provided evidence reporting increased cognitive impairments, impaired hippocampal neurogenesis and increased AD-type pathologies in female, as compared to male, transgenic amyloid precursor protein × presenilin 1 (APPxPS1) mouse models of AD (e.g., [87,88]). Animal studies have also provided evidence that depletion of sex steroids in female rodents by ovariectomy promotes AD-like pathogenesis (e.g., increased brain levels of soluble β-amyloid (Aβ) protein) [89].

Sex also appears to modulate the impact of genetic risk factors in the aetiology of AD. For example, the *APOE* gene risk allele (*ε4*), the strongest known genetic risk factor for late-onset (i.e., 65+) AD, may confer differentially higher risk for AD conversion in females as compared to males [90,91], particularly in younger females [92]. A recent meta-analysis found a stronger association between *APOE-ε4* and CSF tau levels in females compared with males, particularly among amyloid positive females [91]. Estrogen-level changes following menopause were hypothesised to underlie this sex difference in tau, potentially through direct mechanisms such as estradiol’s capacity to protect against tau hyperphosphorylation or indirectly via estrogen’s capacity to reduce beta-amyloid toxicity [91]. Other genetic variants have also shown sex-specific effects on risk and progression of AD, such as the *Met66* allele of the Brain Derived Neurotrophic Factor (*BDNF*) gene, which has been associated with an increased risk of AD in females as compared to males [93].

## 7. Hormonal Treatments for Cognitive Impairments

While the evidence supporting a link between estrogen depletion and risk for AD appears relatively consistent [89], the influence of hormone therapies (HT) containing estrogen to enhance cognition (e.g., during menopause) or reduce AD risk is controversial and the results are inconsistent [94]. The Women’s Health Initiative Memory Study (WHIMS), an ancillary study of the Women’s Health Initiative (WHI) hormone therapy trials, was conducted a decade ago and concluded that Premarin (estrogen plus a synthetic progestin) was associated with increased risk for dementia and reduced cognitive functioning based on the Modified Mini-Mental State (3MS) [95]. The WHI and WHIMS fuelled substantial public and academic debate about risks (cancer, cardiovascular and dementia risks) associated with HT, however, the initial interpretation of the WHIMS findings have since been criticized based on a number of issues. These include the idea of a healthy cell bias, which suggests estrogens will be neuroprotective in a healthy environment but not in a disease environment. The WHIMS study included participants that had health disorders, and several studies have since suggested that the efficacy of HT may be contingent on the health status of individuals, whereby the estrogen’s neuroprotective effects may depend on having healthy neurons [96,97]. A second area of critique relates to the ‘critical window’ or ‘timing’ hypothesis [96,98,99], which proposes that HT is only effective when initiated early in menopause or just prior to menopause (the women in the WHIMS study averaged 15 years post-menopause). The type of HT is also considered relevant to the potential for cognitive benefits and/or risks. The HT in the WHIMS study was Premarin which is approximately 50% sulphated estrone and 1% estradiol, plus a synthetic progesterone. This is relevant as 17β-estradiol has positive effects on cognition, whereas estrone has been associated with negative effects on cognition [97].

In the last decade, several observational and randomised controlled trials have provided some evidence to suggest that HT may reduce the risk of AD and that early use is protective and later use is either not protective or detrimental [99]. However, two recent clinical trials specifically investigating the timing hypothesis in healthy postmenopausal females have failed to provide support to suggest a greater benefit is associated with earlier use of HT. The Kronos Early Estrogen Prevention Study (KEEPS) assessed cognitive outcomes in a total of 662 early postmenopausal females following four years of either oral conjugated equine estrogens (CEE) plus micronized progesterone or transdermal estradiol plus micronized progesterone. Results found that there was no cognitive benefit for either HT, as compared to placebo, on any of the cognitive domains measured [100]. The Early versus Late Intervention Trial with Estradiol (ELITE) [101] compared approximately five years of oral micronized 17β-estradiol (with vaginal micronized progesterone gel) to placebo in women stratified as ‘early’ or near menopause (within 6 years of a final menstrual period) or ‘late’ (more than 10 years postmenopause). As compared to the placebo, estradiol initiated within 6 years of menopause did not affect verbal memory, executive functions, or global cognition differently to estradiol commenced 10 or more years following menopause [102]. In summary, it appears that there is a lack of evidence supporting the timing hypothesis for enhancing cognition in postmenopausal women. Of note, the findings from these two large clinical trials also suggested that there were no adverse cognitive outcomes associated with HT (and neither trial evaluated dementia risk). Further investigations into HT timing and the potential to lower dementia risk is required.

In relation to hormone types, continuous, combined CEE/synthetic medroxyprogesterone acetate (MPA) has not been associated with cognitive benefits (regardless of timing), whereas estrogen alone (including studies using ultra-low-dose transdermal estradiol as well as CEE), as well as estradiol valerate combined with diongest or norehindrone may result in cognitive benefits (as reviewed by [99]). The type of progestogenic compound (progesterone versus different synthetic progestins) appears very relevant to the cognitive impact [18,103]. The synthetic MPA is the most commonly used progestin in HT regimens and current evidence suggests that MPA, in contrast to progesterone, has a negative impact on markers of neuroprotection and neurogenesis [104]. While there are few studies of unopposed progesterone treatment in postmenopausal women, a small 12-week trial of daily 200 mg progesterone was associated with an improvement in verbal working memory [103]. There have also been a series of observational and clinical trials using testosterone treatment in postmenopausal women, with results suggesting low concentrations of testosterone treatment (to replicate premenopausal levels) are associated with improvements in verbal learning and memory (see [51] for review). Testosterone trials have also been conducted in males to assess the efficacy of testosterone treatment in older men (aged 65 plus) with memory impairment and low testosterone levels; however, current evidence suggests testosterone treatment in older males is not associated with improved memory or other cognitive functions [105]. While current evidence does not justify the use of HT of any type to specifically enhance cognition or reduce dementia risk, further investigation is warranted to investigate the potential for estrogens, progesterones and testosterone to determine their individual and combined capacity to enhance cognition and reduce AD risk.

The development of selective estrogen receptor modulators (SERMs), such as raloxifene, provide an estrogen therapy with mixed agonist/antagonist properties, thus avoiding some of the adverse risks that have been associated with estradiol therapy (e.g., see [106]). Raloxifene has antagonist effects on the estrogen receptor in the breast and uterus, while maintaining agonistic effects on the estrogen receptors in bone and brain tissue [107]. Raloxifene, currently approved for use in postmenopausal women with osteoporosis, has been associated with mixed effects on cognition, potentially reflecting variations in methodology, dose and study populations [108]. A recent systematic review on the effects of raloxifene on cognition in postmenopausal women concluded that a dose of 120 mg/day may have some benefit for cognition in relation to aging and risk of cognitive decline [108]. Again, further research into the role of SERMs as cognitive enhancers and their role in reducing AD risk is warranted.

## 8. Sex-Based Lifestyle Risks and Interventions for AD

In addition to the biological explanations for the observed sex differences in cognitive decline and AD risk is a range of related health and lifestyle factors, which can be associated with gender differences due to sociological factors as well as sex differences. An increased cognitive reserve/resilience, that is, a higher level of education/occupation and greater engagement in cognitive activities, is considered a protective factor for AD. Education, a key mediator of cognitive reserve, has historically been associated with gender differences and the educational attainment for the older population of today may explain some gender differences between the level of cognitive reserve [83,109]. Stress and dysregulation of the stress-axis (hypothalamic-pituitary-adrenal (HPA) axis) has been associated with increased risk of AD, and sex differences in different aspects of stress responses (as reviewed in [110]), as well as modulation of the HPA axis by sex hormones may contribute to sex differences in AD [109]. There are also sex differences in metabolic and vascular factors which may also contribute to sex differences in AD [109].

A longitudinal study of non-demented older adults who were identified as carrying two AD risk genes (*APOE* and *Clusterin*) were assessed for sex-specific resilience factors [111]. While some resilience factors applied to both men and women, such as younger age, higher education, grip strength and everyday novel cognitive activity, there were a number of sex-specific resilience factors. For women, predictors of resilience related to demographic (living status and marital status), functional biomarkers (peak expiratory flow and pulse pressure), subjective health, mobility and lifestyle (e.g., volunteering and social visits) categories. For men, only fewer depressive symptoms were an important predictor. Extending these findings, the authors provided suggestions for sex-specific lifestyle targets. For example, women with AD genetic risk may benefit from interventions that improve cardiovascular, respiratory and mobility functions, while men with AD genetic risk factors may benefit most from interventions that target physical fitness, treatment of depressive symptoms and everyday cognitive activities. Further research investigating sex-specific risk and resilience factors may help to develop a better understanding of sex-specific modifiable lifestyle risk-reduction practices.

## 9. Conclusions

Further research is warranted to better understand the role of sex differences, sex hormones, gender and psychosocial factors that influence cognitive functioning, particularly in relation to cognitive decline and risk of AD. From a research perspective, it is important to stratify results by sex (rather than controlling for sex) to continue to investigate how sex and sex hormones contribute to cognitive health and cognitive decline. Sex hormones (estrogens, androgens and more recently luteinizing hormone) are influential in maintaining neuronal health and promoting neuronal cascades that underpin cognitive processes. As the potential for therapeutic modulation of the endocrine system advances, it is essential we understand how sex hormones influence cognitive functioning in AD. Much can be gained from advancing research in this field. Specifically, determining which hormonal pathways are most relevant to cognitive decline in AD. There is also the capacity for sex-related targeting of modifiable risk factors. A sex-specific focus of cognitive decline and AD is still not mainstream. Given the growing evidence supporting a role for sex hormones in cognitive functioning, combined with the many documented sex differences associated with AD, it appears that much can be gained from advancing research in this field.
